# Kimma: flexible linear mixed effects modeling with kinship covariance for RNA-seq data

**DOI:** 10.1093/bioinformatics/btad279

**Published:** 2023-05-04

**Authors:** Kimberly A Dill-McFarland, Kiana Mitchell, Sashank Batchu, Richard Max Segnitz, Basilin Benson, Tomasz Janczyk, Madison S Cox, Harriet Mayanja-Kizza, William Henry Boom, Penelope Benchek, Catherine M Stein, Thomas R Hawn, Matthew C Altman

**Affiliations:** Division of Allergy and Infectious Diseases, Department of Medicine, University of Washington, 750 Republican St, Seattle, WA 98109, United States; Division of Allergy and Infectious Diseases, Department of Medicine, University of Washington, 750 Republican St, Seattle, WA 98109, United States; Department of Biology, University of California San Diego, 9500 Gilman Dr, La Jolla, CA 92093, United States; Division of Allergy and Infectious Diseases, Department of Medicine, University of Washington, 750 Republican St, Seattle, WA 98109, United States; Division of Allergy and Infectious Diseases, Department of Medicine, University of Washington, 750 Republican St, Seattle, WA 98109, United States; Systems Immunology Division, Benaroya Research Institute, 1201 Ninth Avenue, Seattle, CA 98101, United States; Systems Immunology Division, Benaroya Research Institute, 1201 Ninth Avenue, Seattle, CA 98101, United States; Division of Allergy and Infectious Diseases, Department of Medicine, University of Washington, 750 Republican St, Seattle, WA 98109, United States; Department of Medicine, School of Medicine, Makerere University, PO Box 7072, Kampala, Uganda; Department of Medicine, Case Western Reserve University, 10900 Euclid Ave, Cleveland, OH 44106, United States; Department of Population & Quantitative Health Sciences, Case Western Reserve University, 10900 Euclid Ave, Cleveland, OH 44106, United States; Department of Population & Quantitative Health Sciences, Case Western Reserve University, 10900 Euclid Ave, Cleveland, OH 44106, United States; Division of Allergy and Infectious Diseases, Department of Medicine, University of Washington, 750 Republican St, Seattle, WA 98109, United States; Division of Allergy and Infectious Diseases, Department of Medicine, University of Washington, 750 Republican St, Seattle, WA 98109, United States; Systems Immunology Division, Benaroya Research Institute, 1201 Ninth Avenue, Seattle, CA 98101, United States

## Abstract

**Motivation:**

The identification of differentially expressed genes (DEGs) from transcriptomic datasets is a major avenue of research across diverse disciplines. However, current bioinformatic tools do not support covariance matrices in DEG modeling. Here, we introduce kimma (Kinship In Mixed Model Analysis), an open-source R package for flexible linear mixed effects modeling including covariates, weights, random effects, covariance matrices, and fit metrics.

**Results:**

In simulated datasets, kimma detects DEGs with similar specificity, sensitivity, and computational time as limma unpaired and dream paired models. Unlike other software, kimma supports covariance matrices as well as fit metrics like Akaike information criterion (AIC). Utilizing genetic kinship covariance, kimma revealed that kinship impacts model fit and DEG detection in a related cohort. Thus, kimma equals or outcompetes current DEG pipelines in sensitivity, computational time, and model complexity.

**Availability and implementation:**

Kimma is freely available on GitHub https://github.com/BIGslu/kimma with an instructional vignette at https://bigslu.github.io/kimma_vignette/kimma_vignette.html.

## 1 Introduction

Transcriptomics provide a powerful method to capture genome-wide RNA expression at relatively low costs. Methods such as microarray and RNA-seq allow for in-depth assessment across populations and time scales relevant to research in human health, disease, ecology, and others. Transcriptomics studies tend to have experimental designs with many more measures (e.g. genes) than observations (e.g. subjects or libraries). Thus, subsequent analyses require multiple comparison correction, which can obscure true biological results in an effort to reduce Type I error (Korthauer et al. 2019). In addition, numerous or complex covariates can be necessary to correct for diverse populations, and designs can include repeated measures, paired sampling, or blocking. Together, these features require diverse statistical methods and robust model fit assessments to achieve accurate transcriptomic results.

Presently, there are a variety of analysis pipelines for the identification of differentially expressed genes (DEGs) in bulk transcriptomic datasets (Conesa et al. 2016). DEGs represent one of the major results from RNA-seq experiments and drive discoveries in this space. While current DEG analysis tools can accommodate a range of models, none support the inclusion of complex random effects like covariance matrices. These matrices consist of more than one measure per observation, such as pairwise comparisons, and thus, cannot be modeled as fixed or simple random effects in current software. In addition, covariance random effects are distinct from the challenges of covariance within transcriptomic data which have been explored in several recent studies using coexpressed genes (Kochan et al. 2021) or gene sets (Chang et al. 2017; Qayed and Han 2021).

Among current parametric DEG pipelines, limma (Ritchie et al. 2015), DESeq2 (Love et al. 2014), and edgeR (Robinson et al. 2010) are the most popular, each with well over 10 000 citations since their publication. Limma utilizes simple linear regression and empirical Bayes statistics to assess differential gene expression. This R package also supports gene-specific weights to account for library quality (Law et al. 2014) and a pseudorandom effect to account for paired sample designs or repeated measures. Others have expanded on the limma framework with dream in the VariancePartition R package to incorporate true random effects to account for paired designs more accurately as well as support multiple blocking variables in complex mixed effects designs (Hoffman and Roussos, 2021). In contrast, DESeq2 and edgeR employ negative binomial regression to assess differential expression. This statistical framework does not require gene-level weights but also does not support random effects or paired designs. Overall, these methods support many RNA-seq study designs but fail to incorporate those with potential covariance effects. Moreover, current software provides minimal fit metrics for assessment of a “best fit” model.

One potential covariance effect of interest in transcriptomic research is kinship, a summative measure of genetic relatedness that estimates recent family structure while correcting for long-term population structure (Manichaikul et al. 2010). Previous transcriptomic studies have estimated relatedness through coarse measures such as family groups; however, more granular relatedness measures are needed since only a subset of single nucleotide polymorphisms (SNPs) correlate with gene expression and heritability varies across the genome (Price et al. 2011). Moreover, pedagogies assume perfect Mendelian genetics and are subject to errors as a result of incorrect or incomplete family histories. Kinship determined from genetic sequencing provides a better estimate of relatedness as it is directly measurable and not reliant on historical memory. Moreover, kinship has been shown to describe recent population relatedness beyond immediate family groups more accurately than social constructs like race and ethnicity (Collins et al. 2003). Thus, kinship may improve DEG analyses in both related and unrelated cohorts by correcting for underlying genetic differences that confound true signals of interest.

Here, we present kimma for kinship in mixed model analysis to support DEG analyses including covariance random effects. Kimma is an open-source R package that provides flexible linear mixed effects modeling for bulk RNA-seq data including univariate, multivariate, random, and covariance random effects as well as gene-level weights. Building on well-tested statistical packages in R such as stats (R Core Team 2022) and lme4 (Bates et al. 2015), kimma utilizes a single function, kmFit, for modeling, ensuring consistent syntax, inputs, and outputs. Moreover, kimma provides *post hoc* pairwise tests, model fit metrics like Akaike information criterion (AIC), and fit warnings on a per gene basis. Kimma’s outputs can also be integrated into our sister R packages in the BIGverse for visualization of results as well as gene set analyses.

## 2 Materials and methods

### 2.1 Data acquisition and generation

#### 2.1.1 Tuberculosis dataset

Study recruitment (Stein et al. 2019) and RNA-seq experiments (Simmons et al. 2018, 2022) were previously described. Briefly, household contacts of individuals with active pulmonary tuberculosis were followed for 8–10 years and classified as persistently TST-/IGRA- resisters (RSTR) or latent tuberculosis infection (LTBI). Monocytes were extracted from whole blood and cultured without stimulation (media) or with *Mycobacterium tuberculosis* strain H37Rv (Mtb; MOI 1.0, 6 h). Bulk RNA sequencing was performed using Illumina 50 bp paired-end sequencing, and sequences were aligned to GRCh38 with STAR (Dobin et al. 2013). Low abundance genes were removed if they did not reach at least 1 count per million (CPM) in 5% of samples in the original dataset. Genetic kinship was determined from Illumina MEGAEX array data and methods as described in McHenry et al. (2021). Briefly, 63 812 high-quality SNPs were obtained from the subset of the Ugandan cohort used in this study (minor allele frequency > 5%, Hardy–Weinberg disequilibrium *P* < 1E−6, call rate > 95%, linkage disequilibrium *R*^2^ < .1). Kinship was calculated from SNPs using the robust KING method corrected for two population principal components.

#### 2.1.2 Simulated data generation

In order to maintain the paired sample design, 100 simulated DEGs datasets were created from the uninfected, media samples from the RSTR/LTBI tuberculosis dataset. In total, 1000 random genes were selected and copied for simulated samples. Then, 50 DEGs were created at each fold change of 1%, 5%, 10%, 50%, and 100%, and random error of ±5% was introduced to all genes.

### 2.2 Statistical models

#### 2.2.1 Limma and dream models

Gene expression was modeled as trimmed mean of means (TMM) normalized log_2_ CPM. For limma (v3.50.3; Ritchie et al. 2015), linear models were fit, and *P*-values were estimated using the empirical Bayes method. Pseudo-paired sample design was estimated using mean correlation of expression between paired samples across all genes (duplicateCorrelation). For dream (variancePartition v1.24.0; Hoffman and Roussos 2021), only paired sample designs were run as its unpaired designs are equivalent to limma. Linear mixed effects models were fit blocked by donor, and *P*-values were estimated using the empirical Bayes method. Where specified, gene-level quality weights were calculated using voomWithQualityWeights for limma or dreamWithQualityWeights for dream and incorporated in the models.

#### 2.2.2 DESeq2 and edgeR models

Gene expression was modeled as unnormalized counts as recommended by the software. For DESeq2 (1.34.0; Love et al. 2014), negative binomial general linear models including total library size and estimated dispersion were fit, and *P*-values were estimated using the Wald test. For edgeR (3.38.4; Robinson et al. 2010), quasi-likelihood negative binomial general linear models including total library size and estimated dispersion were fit, and *P*-values were estimated using the *F*-test. For both, pseudo-paired sample design was estimated by including donor as the first main effect term. DESeq2 and edgeR do not support gene-level quality weights.

#### 2.2.3 Kimma models

Gene expression was modeled as TMM normalized log_2_ CPM. For kimma (v1.2.0), linear models were fit, and *P*-values were estimated using restricted maximum likelihood. Simple linear regression was performed with base R stats::lm (R Core Team 2022), mixed effects for paired sample designs with lme4::lmer (Bates et al. 2015), and mixed effects for paired sample designs with kinship with lme4qtl::relmatLmer (Ziyatdinov et al. 2018). As with limma, gene-level quality weights calculated using voomWithQualityWeights were incorporated for some models. Though not applicable in these analyses, kimma can also fit pairwise contrasts within main model terms using estimated marginal means in the emmeans package (Lenth 2022).

### 2.3 Data analysis

#### 2.3.1 Simulated data analysis

Simulated DEGs were modeled against their corresponding media samples. Within each of 100 simulated datasets, sensitivity was defined as true positive/(true positive + false negative) and specificity as true negative/(true negative + false positive). Area under the curve (AUC) was calculated for 1 − specificity by sensitivity at *P*-value cutoffs in increments of 0.001 from 0 to 0.01 plus increments of 0.01 from 0.01 to 1. Time trials were run on Amazon Web Services (48 vCPU, 192 GB RAM) with one or six processors as appropriate and nothing else running.

#### 2.3.2 Tuberculosis data analysis

In total, 13 972 pass-filter genes (see Simmons et al. 2022) were modeled for media versus Mtb-infected in paired LTBI samples (*n* = 92) as well as RSTR versus LTBI in unpaired Mtb-infected samples (*n* = 98). Analyses were completed on the entire dataset as well as subsets of unrelated (kinship < 0.125, *n* = 55, 62) and related samples (at least one kinship > 0.125, *n* = 37, 36). Genes found to be significant for Mtb versus media in the related subset with kinship correction were extracted from genetic data with SNPs found on both the MEGAEX and Omni chip (McHenry et al. 2021). In total, 3382 of these 8525 DEGs had at least 1 pass-filter SNP within the gene transcript region (*cis*) to yield 10 020 unique SNP–DEG pairs. Each SNP–DEG pair was modeled as an expression quantitative loci (eQTL) using the interaction of Mtb infection and genotype. Significant genes and eQTLs were defined at false discovery rate [FDR] < 0.05. Model fit was measured by AIC.

## 3 Results

**Figure 1.**
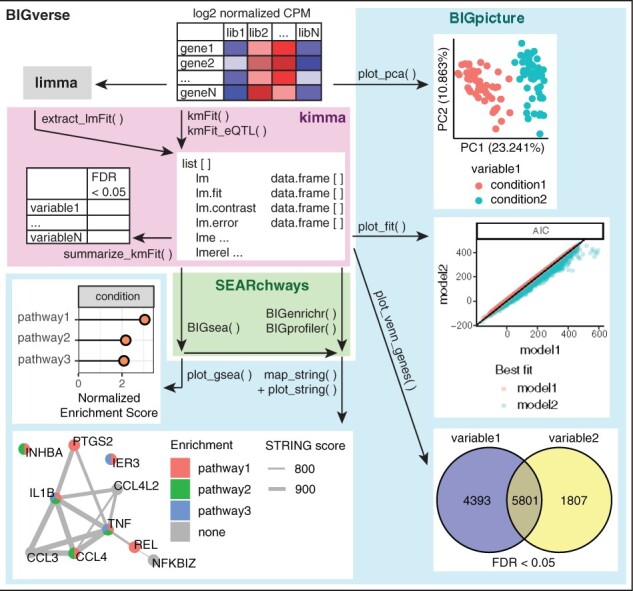
BIGverse workflow. Log2 normalized CPM gene expression is input into the BIGverse pipeline. Prior to modeling, variables of interest can be explored by PCA using BIGpicture. Gene expression is then modeled in kimma with kmFit using simple linear (lm), linear mixed effects (lme), and/or lme with kinship relatedness (lmerel). Each model results in up to four data frames: estimates and significance, goodness of fit metrics, pairwise contrasts, and errors for genes that failed fitting. Model fit is assessed by metrics, such as AIC, which can be plotted in BIGpicture. Significant genes are summarized in kimma and plotted using Venn diagrams in BIGpicture. The SEARchways package is used for pathway analyses such as gene set enrichment analysis (GSEA) with kmFit model estimates of fold change and hypergeometric pathway enrichment of significant genes. Significant pathways are visualized as well as annotated to specific genes in STRING networks using BIGpicture. In addition, kimma provides functionality to import limma results for use in parallel analyses and visualization.

The kimma R package provides flexible linear modeling of RNA-seq data with a single function, kmFit. This package is open-source and freely available on GitHub (https://github.com/BIGslu/kimma). Kimma fits linear and/or linear mixed effects models incorporating gene-level weights, covariates, and/or random effects. In addition, kimma provides easy comparison of model fits with metrics such as AIC, BIC, and *R*^2^ as well as downstream analyses including summary tables and eQTL. The kimma outputs feed directly into our related packages such as plotting in BIGpicture and gene set analysis in SEARchways. Collectively, these R packages work together in the meta-package BIGverse (Fig. 1).

### 3.1 Benchmarking kimma using simulated data

#### 3.1.1 Kimma performs as well as current methods for DEG analysis without kinship

**Figure 2.**
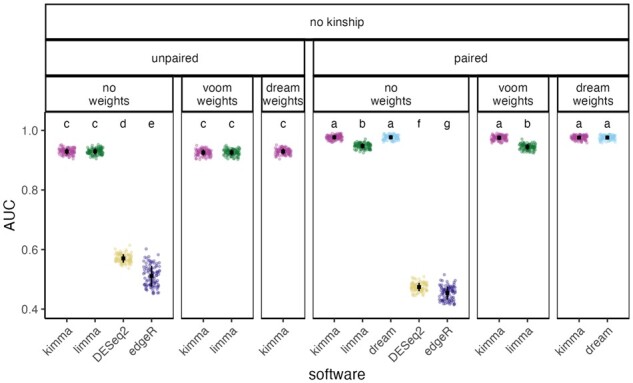
DEGs detected across linear models and software. AUC was calculated from curves of 1 − specificity to sensitivity for *P*-value cutoffs from 0 to 1 in 100 simulated datasets. When available, models were corrected for gene-level weights (kimma, limma, dream) and/or paired design (kimma, limma, dream, DESeq2, edgeR) as indicated by panel headers. Black squares indicate means with standard deviation bars. Across all panels, letters indicate statistically different groups, Tukey FDR < 0.05, in order from highest AUC (a) to lowest (g). Points are colored by software package.

To test kimma’s performance, we constructed simulated test datasets from previously published RNA-seq data which included two groups [resister (RSTR) and LTBI] and two conditions [media and Mtb infection] in a household-based cohort of individuals with varying degrees of relatedness (Simmons et al. 2018, 2022). Modeling was performed in kimma, limma, dream, DESeq2, and edgeR using 100 simulated datasets of 250 DEGs with fold changes from 1% to 100% and 750 non-DEGs (Supplementary Fig. S1A). In unpaired model designs, kimma and limma resulted in similar sensitivity and specificity (mean AUC 0.93; Fig. 2). DESeq2 and edgeR’s negative binomial models performed significantly worse than all unpaired kimma or limma models (mean AUC 0.57, 0.51, FDR < 0.05). In paired designs, kimma’s random effect and dream’s repeated measures (mean AUC 0.98) slightly outperformed limma’s duplicateCorrelation pseudorandom effect (mean AUC 0.95) and greatly outperformed DESeq2 and edgeR’s fixed effect for donor (mean AUC 0.47, 0.45, FDR < 0.05). Within a model type and software, gene-level weights had no effect on DEG detection.

#### 3.1.2 Kimma’s multiprocessor functions achieve runtimes similar to limma

**Figure 3.**
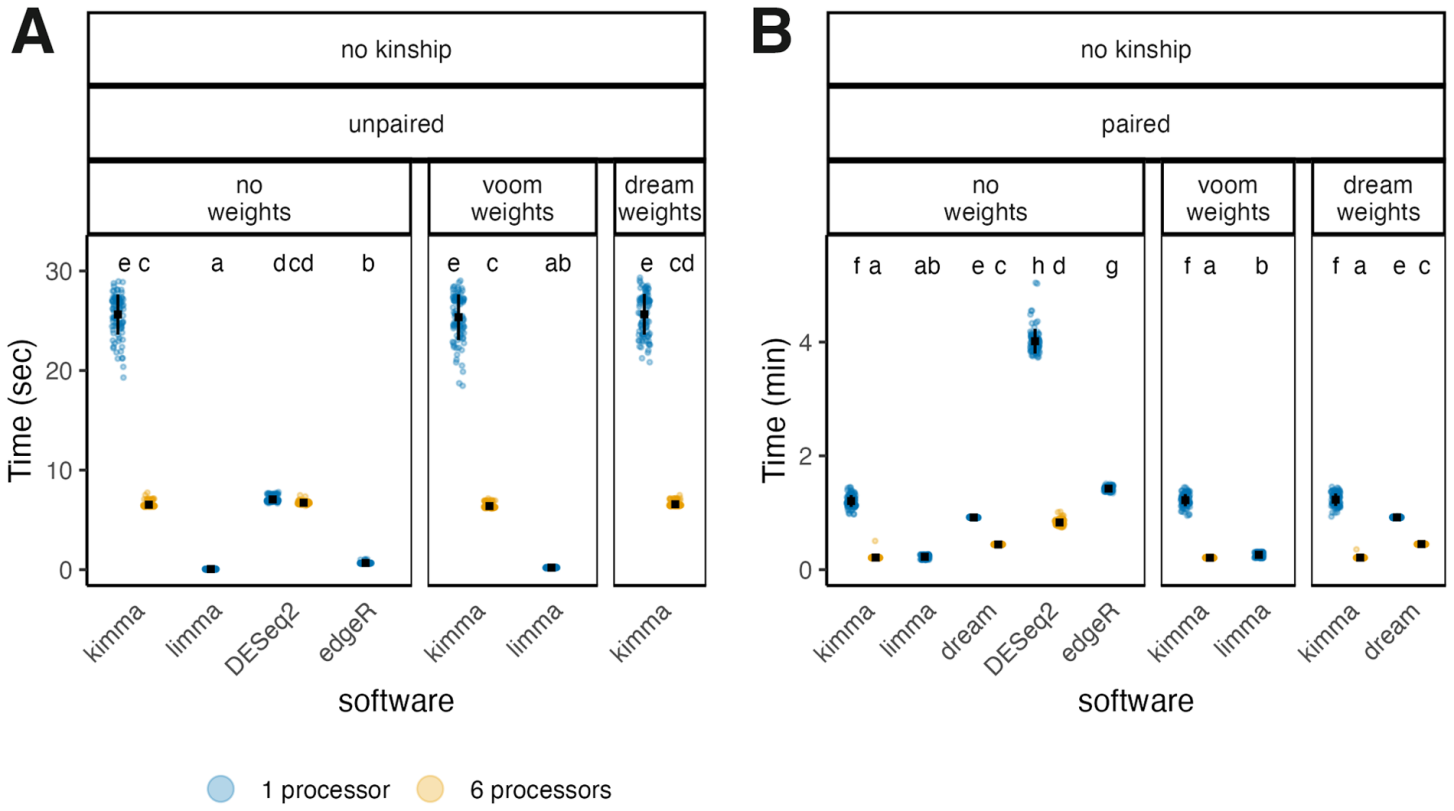
Runtime to analyze simulated RNA-seq data across linear models and software. (A) Unpaired models without random effects. Time in seconds. (B) Paired models with donor as a random effect. Time in minutes. Models were run for 100 simulated datasets of 92 samples and 1000 genes. When available, models were corrected for gene-level weights (kimma, limma, dream) and/or paired design (kimma, limma, dream, DESeq2, edgeR) as indicated by panel headers. Multiprocessor usage is shown for kimma, dream, and DESeq2 at one (blue) or six processors (orange). Limma and edgeR do not have this option. Black squares indicate means with standard deviation bars. Within (A) or (B) separately, letters indicate statistically different groups, Tukey HSD FDR < 0.05, in order from shortest time (a) to longest (e or h).

Multiple processor functionalities in kimma, dream, and DESeq2 were compared across simulated datasets (Supplementary Fig. S1A) on six processors as this is a reasonable load for standard 8-core laptops. In paired model designs, kimma runtime linearly decreased from 72 s on one processor to 12 s on six processors (Fig. 3B). Kimma’s six-processor speed was as fast or slightly faster than all other software in paired designs (FDR < 0.05). In contrast, dream was slower and did not reduce time linearly, taking 55 s on one processor and 26 s on six processors. DESeq2 and edgeR were the slowest software for paired designs on one processor, though these methods use donor as a fixed effect, thus increasing model complexity compared with other methods. In contrast, limma outperformed all other software with edgeR as a close second for speed in unpaired designs (Fig. 3A).

### 3.2 Benchmarking kimma using real-world data

#### 3.2.1 Kimma provides additional functionalities at no cost to computational time

We next used full-size data analysis to test whether kimma can incorporate multiple model types and other features while keeping computational time low. In a dataset of 13 972 protein-coding genes and 98 samples, model fitting and significance estimation takes <90 s (Supplementary Fig. S2). The addition of fit metric calculation, gene-level weights, covariates, or interaction terms has no effect on total processing time. Adding pairwise contrasts between multilevel variables or interaction terms, paired sample design as a random effect, or kinship as a covariance random effect increases computational time to between 3 and 4 min for this dataset. Combining kimma’s features increases processing time almost linearly as a model with all seven features increased time by a factor of six and running three models at once takes roughly the sum of time for the three models run separately.

#### 3.2.2 Kimma detects more DEGs in real-world paired model designs

We next compared DEG detection in real-world data. In a dataset of Mtb infected versus media controls in LTBI and RSTR donors (Simmons et al. 2022), DEGs were modeled using the best performing methods based on simulated data analyses (Fig. 2). Thus, kimma and dream were compared for paired designs (Fig. 2, Group a) while kimma and limma were compared for unpaired designs (Fig. 2, Group c).

**Figure 4.**
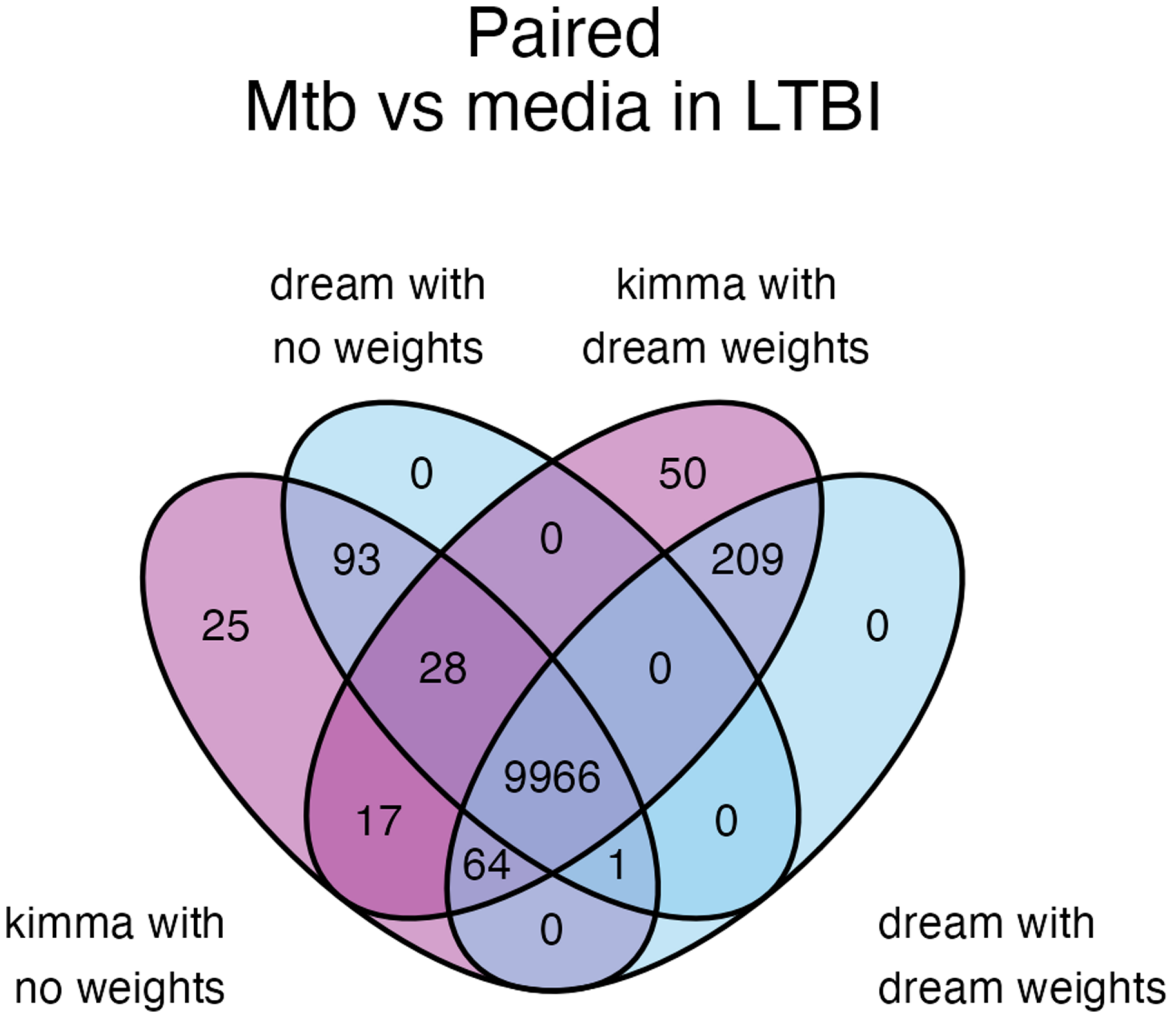
Real-world RNA-seq data analysis across paired models and software. Using the best performing models from simulated data, real-world data was assessed for the impacts of Mtb infection in paired designs (Fig. 2, Group a). Venn diagrams show total DEGs.

For paired Mtb infected and media samples (Supplementary Fig. S1B), the majority of DEGs were detected by all models, and gene-level weights resulted in the largest addition of 209 DEGs regardless of software (FDR < 0.05; Fig. 4). Overall, kimma with weights resulted in the most DEGs with 95 more DEGs than dream with weights, of which 50 were specific to only the kimma with weights method. At an FDR of 0.05, simulated data analysis showed kimma to have minimally but consistently higher sensitivity (max delta sensitivity 0.02) when compared with dream (Supplementary Table S1). This supports the detection of more true-positive DEGs by kimma in the Mtb analysis compared with dream. In contrast, an unpaired analysis of LTBI versus RSTR (Supplementary Fig. S1C) resulted in six DEGs identified by all models and software (FDR < 0.05). This is further supported by the unpaired simulated analysis where differences in sensitivity were very low and did not always support the same model (max delta sensitivity 0.008, Supplementary Table S1).

### 3.3 Novel kimma models and features

#### 3.3.1 Kinship improves model fit and DEG detection in paired, genetically related datasets

**Figure 5.**
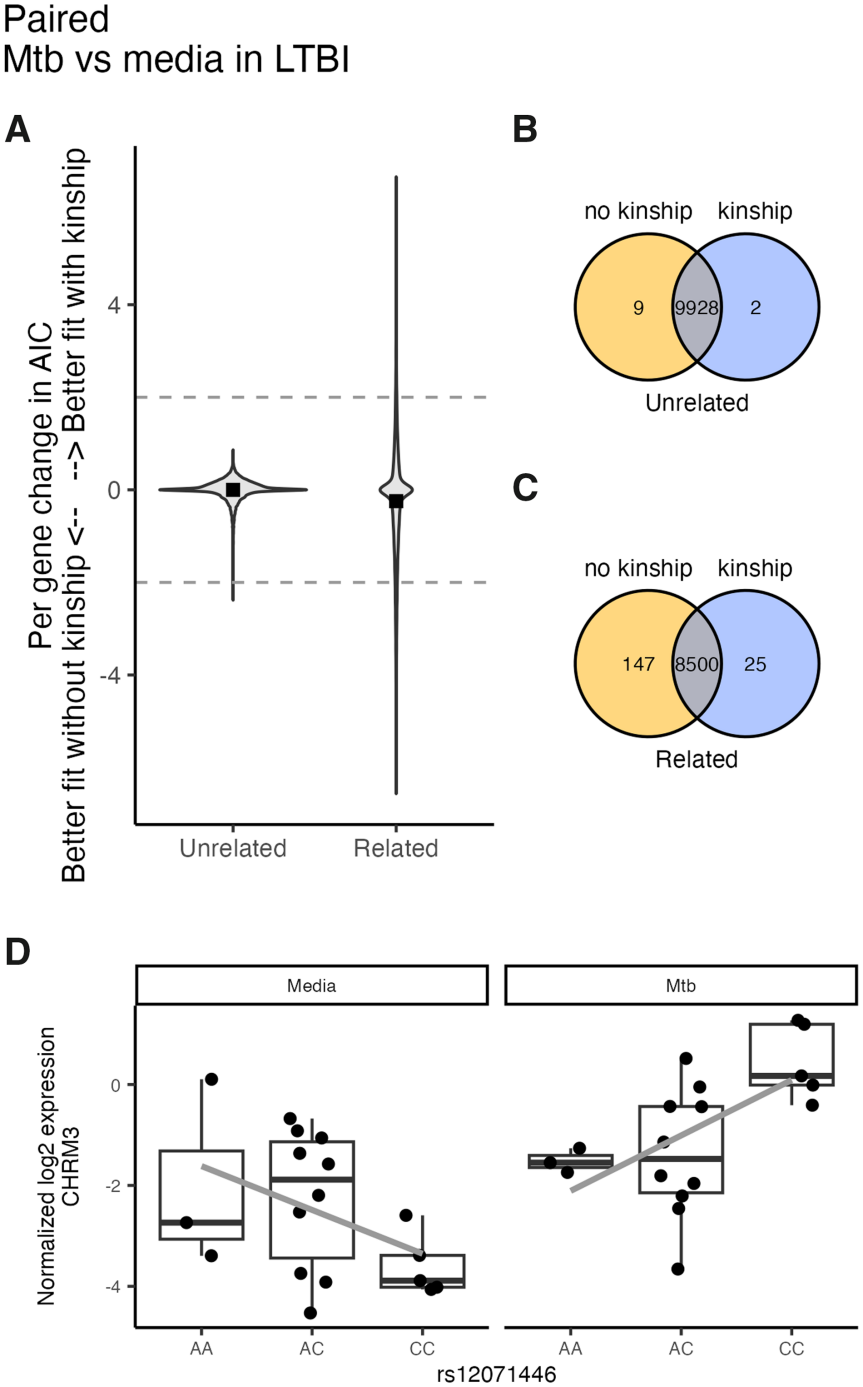
Paired design model fit and DEGs in genetically unrelated and related datasets. LTBI individuals were subset into unrelated and related subsets at a kinship cutoff of 0.125. Subsets were modeled for Mtb-infected versus media expression. (A) Model fit was assessed by AIC. Change in AIC was calculated per gene for the model without kinship minus the model with kinship. Black squares indicate means. Dashed lines indicate minimal change in AIC (*y* = −2 or 2). DEGs were defined at FDR < 0.05 in the (B) unrelated and (C) related subsets. (D) Expression of the top Mtb: SNP cis eQTL from kinship DEGs in the related subset.

Unlike other RNA-seq analysis packages, kimma allows incorporation of a covariance random effect such as pairwise genetic kinship. Here, the Mtb versus media and RSTR versus LTBI data were split into unrelated (kinship < 0.125) and related subsets (at least one kinship > 0.125) (Supplementary Fig. S1B and C) to assess the impacts of kinship in linear mixed effects models. In the paired Mtb infected versus media model, the addition of kinship among unrelated individuals did not impact model fit (Fig. 5A) with only two genes reaching an absolute delta AIC > 2 (max delta AIC = 2.4). This corresponded with minimal changes in DEG detection as both models identified 99.9% of the same genes at FDR < 0.05 (Fig. 5B).

In contrast, Mtb model fit of related individuals was impacted by kinship (Fig. 5A) with 1247 genes better fit by >2 AIC with kinship (max delta AIC = 6.6) and 518 better fit without kinship (max delta AIC = 6.8). This resulted in changes in DEG detection with 25 new DEGs detected in the kinship model (Fig. 5C). Importantly, 91 of the 147 DEGs only detected without kinship were better fit by the model with kinship according to AIC. Thus, the addition of kinship appears to reduce Type I error by improving fit for a subset of genes.

In the unpaired RSTR versus LTBI design, kinship did not improve model fit with the majority of genes (98%) better fit without kinship in the model for both unrelated and related subsets (max delta AIC = 23; Supplementary Fig. S3A). While more DEGs were detected with kinship in the unpaired, related subset, the majority of these genes (369 of 376) were better fit by AIC without kinship in the model. Thus, the inclusion of kinship in unpaired designs with minimal relatedness may result in higher Type I error due to a poorer fitting unpaired model.

#### 3.3.2 Kimma extends to other applications such as eQTL analysis

Downstream analysis of RNA-seq data often includes expression quantitative trait loci (eQTL), where SNPs are associated with gene expression. Kimma’s kmFit_eQTL function provides the means to test eQTLs with the same flexible modeling and fit assessment features as kmFit. As a test case, we utilized genome-wide SNP data from the same cohort to determine eQTL for DEGs identified in the Mtb-related kinship model. Among the kinship model DEGs (*n* = 8525; Fig. 5C), we identified 10 020 potential within-gene *cis* eQTL in 3382 unique genes. In total, 289 eQTL in 253 unique genes were significant for the interaction of SNP and Mtb infection (FDR < 0.05). The top hit was genotype rs12071446 which was associated with Mtb-induced expression of the CHRM3 gene (FDR = 2.2E−7; Fig. 5D). This analysis framework can be readily applied to any gene(s) of interest to determine genetic regulation of gene expression using the same robust models as DEG detection.

## 4 Discussion

Transcriptomics is utilized in many fields to address diverse scientific questions. Identification of DEGs remains a cornerstone of analysis of these data, and current bioinformatic tools support a range of study designs. Here, we introduce kimma, an open-source R package that expands supported parametric designs to include covariance random effects. In addition, kimma provides robust model fit assessment and integrates with several downstream analysis and visualization functions for a more streamlined RNA-seq analysis pipeline.

Kimma does not present new statistical methods but instead, builds on well-tested linear modeling methods in R. Simple linear models use base R’s lm (R Core Team 2022) and linear mixed effects models use lme4’s lmer (Bates et al. 2015). Both are widely used and have been available in R for nearly 20 years. Models with covariance matrices are a new addition and utilize lme4qtl’s relmatLmer (Ziyatdinov et al. 2018), which builds on the foundation of lme4. While there are several R-based methods for modeling with covariance matrices, lme4qtl allows seamless integration with and comparisons to models from lm and lme4. Kimma then brings these methods together to efficiently run thousands of statistical tests while codifying similar parameters such as gene-level weights and resulting in consistent, organized outputs. These results then easily flow into the BIGverse RNA-seq analysis pipeline including data visualization in BIGpicture, gene set analyses in SEARchways, and eQTL analysis in kimma.

In simulated paired study designs, kimma performs as well as or better than limma (Law et al. 2014; Ritchie et al. 2015), dream (Hoffman and Roussos 2021), DESeq2 (Love et al. 2014), and edgeR (Robinson et al. 2010). Kimma’s true mixed effects modeling results in comparable sensitivity and specificity to dream, both of which outperform limma’s pseudorandom effect with duplicateCorrelation. Kimma outperforms dream with shorter computational time on multiple processors. In fact, kimma’s multiprocessor functionality affords runtimes on the order of minutes for full-size RNA-seq data (∼14 000 genes, 100 samples) even when multiple additional features or models are requested. These results translate to real-world paired analyses where kimma detects more DEGs than comparable models in dream. Our results suggest these are true positives as kimma also resulted in slightly higher sensitivity in simulated DEG detection.

In predefined unpaired study designs, limma remained the preferred software because while limma and kimma had similar simulated DEG detection, limma achieved faster runtimes. However, limma does not support model fit assessment through metrics such as AIC and requires multiple models to run pairwise contrasts. In addition, kimma out-performed DESeq2 and edgeR with significantly better DEG detection. Thus, kimma results in equivalent or better DEG detection along with additional functionalities in unpaired analyses where model selection is on-going or multi-level variables such as interaction terms are of interest.

Kimma also allows for the use of covariance matrix random effects in linear modeling; these random effects are not supported by any other current method. Here, we use pairwise genetic kinship as an example, but any covariance matrix may be used. In paired study designs, kinship improved model fit and reduced Type I error in DEG detection in genetically related individuals. This effect was apparent even though the current dataset contains only sparse relatedness with 1.4% of pairs achieving third degree relatedness or higher. Model fit was not significantly impacted by kinship in unrelated individuals. In addition, kinship did not improve model fit and appeared to increase Type I error in unpaired designs. Thus, paired designs benefit from kinship inclusion and are not negatively impacted when kinship is nonsignificant. Together, these analyses highlight the advantages of kimma’s model fit assessment in RNA-seq analyses as other workflows may have readily accepted more false positive DEGs even if the model was not best fit for the data. Poor model fit can lead to nonreproducible results and wasted resources on downstream experiments pursuing what were originally false positives.

Kimma’s flexible model design can be extended for targeted eQTL analysis. Using a simple model of genotype:Mtb infection corrected for kinship, we identified 289 eQTL among Mtb DEG in related individuals. The purpose of this analysis was not to identify novel Mtb eQTL as a full genetic analysis likely requires further covariate assessment such as population principal components, sex, etc. Instead, this showcases kimma’s ability to model more than just RNA-seq differential expression. Given kimma’s computational time, tools such as GENESIS (Gogarten et al. 2019) or GMMAT (Chen et al. 2019) are preferred for genome-wide eQTL analyses. However, kimma provides robust targeted analysis with the same underlying model as differential gene expression as well as yields fit metrics useful in determining covariate usage on a subset of genes prior to genome-wide GENESIS or GMMAT analyses.

While kimma represents an advance in parametric DEG detection, this remains only one facet of transcriptomic data cleaning and analysis. Though outside the scope of this study, it is also worth noting recent work in nonparametric DEG analysis (Gauthier et al. 2020) as well as advances in transcript-level alignment (Pimentel et al. 2017) which may complement kimma analysis in the future. In addition due to the limited availability of open-source RNA-seq data from paired designs with accompanying covariance matrix random effects like kinship, kimma was applied to only one real-world dataset in these analyses. While simulated datasets provided measurable sensitivity and specificity, other real-world data should be explored to better elucidate the impacts of genetic kinship or other covariances on DEG detection. We expect the use of kinship with transcriptomic data to increase, in particular, as methods to extract genotype information directly from RNA-seq data become more routinely used, thereby not requiring additional biological material or monetary cost (Blay et al. 2019). In addition, while kimma was applied to RNA-seq data in these examples, the package itself is not RNA-seq specific. Any dataset with multiple measures can be coerced into a similar format and modeled using the appropriate parameters. The user need only ensure that the data meet the requirements of the linear model being applied.

## 5 Conclusion

Overall, kimma provides a user-friendly, single function workflow for linear model analysis of RNA-seq data. It further provides model fit and downstream analyses relevant to DEGs identified by these linear models. Users can explore kimma and its sister packages for results visualization and downstream gene set analyses in the BIGverse through open-source vignettes available at https://bigslu.github.io/kimma_vignette/.

## Supplementary Material

btad279_Supplementary_Data

## Data Availability

Access to raw transcriptomic data is available through the NCBI database of Genotypes and Phenotypes (dbGaP) accession phs002445.v1.p1 (https://www.ncbi.nlm.nih.gov/projects/gapprev/gap/cgi-bin/preview1.cgi?GAP_phs_code=C0TPIcCeWtsG6oqY). Access must be approved by the study’s data access committee (see Simmons et al. 2022). All scripts and code for this manuscript are available at: https://github.com/BIGslu/kimma_MS_public. The kimma software is available at: https://github.com/BIGslu/kimma.
