# Supplementary material for: Kimma: flexible linear mixed effects modeling with kinship covariance for RNA-seq data
Source: Bioinformatics. 2023 May 4;39(5):btad279. doi: 10.1093/bioinformatics/btad279 (PMC10182851; doi:10.1093/bioinformatics/btad279)
Supplement: btad279_Supplementary_Data [file btad279_supplementary_data.docx]

**Supplementary material**

kimma: flexible linear mixed effects modeling with kinship covariance for RNA-seq data

**Table S1.** Simulated data set analysis sensitivity and specificity at FDR < 0.05. Mean true positive (TP), false positive (FP), false negative (FN), and true negative (TN) detection of differentially expressed genes (DEGs) in simulated data sets of 250 DEG and 750 non-DEG. Sensitivity = TP / (TP + FN) and specificity = TN / (TN + FP).

| paired | weights_type | software | cutoff | TP | FP | FN | TN | sensitivity | specificity |
| --- | --- | --- | --- | --- | --- | --- | --- | --- | --- |
| unpaired | no weights | kimma | 0.05 | 147.28 | 0.02 | 102.72 | 749.98 | 0.58912 | 0.99997333 |
| unpaired | no weights | limma | 0.05 | 147.34 | 0.01 | 102.66 | 749.99 | 0.58936 | 0.99998667 |
| unpaired | no weights | DESeq2 | 0.05 | 81.13 | 36.4 | 168.87 | 713.6 | 0.32452 | 0.95146667 |
| unpaired | voom weights | kimma | 0.05 | 150.73 | 0.06 | 99.27 | 749.94 | 0.60292 | 0.99992 |
| unpaired | voom weights | limma | 0.05 | 150.8 | 0.06 | 99.2 | 749.94 | 0.6032 | 0.99992 |
| unpaired | dream weights | kimma | 0.05 | 149.37 | 0.02 | 100.63 | 749.98 | 0.59748 | 0.99997333 |
| paired | no weights | kimma | 0.05 | 224.93 | 10.73 | 25.07 | 739.27 | 0.89972 | 0.98569333 |
| paired | no weights | limma | 0.05 | 208.77 | 14.35 | 41.23 | 735.65 | 0.83508 | 0.98086667 |
| paired | no weights | dream | 0.05 | 223.76 | 8.98 | 26.24 | 741.02 | 0.89504 | 0.98802667 |
| paired | no weights | DESeq2 | 0.05 | 168.1 | 539.98 | 81.9 | 210.02 | 0.6724 | 0.28002667 |
| paired | voom weights | kimma | 0.05 | 225.22 | 14.52 | 24.78 | 735.48 | 0.90088 | 0.98064 |
| paired | voom weights | limma | 0.05 | 210.44 | 22.38 | 39.56 | 727.62 | 0.84176 | 0.97016 |
| paired | dream weights | kimma | 0.05 | 225.32 | 13.58 | 24.68 | 736.42 | 0.90128 | 0.98189333 |
| paired | dream weights | dream | 0.05 | 223.95 | 11.57 | 26.05 | 738.43 | 0.8958 | 0.98457333 |


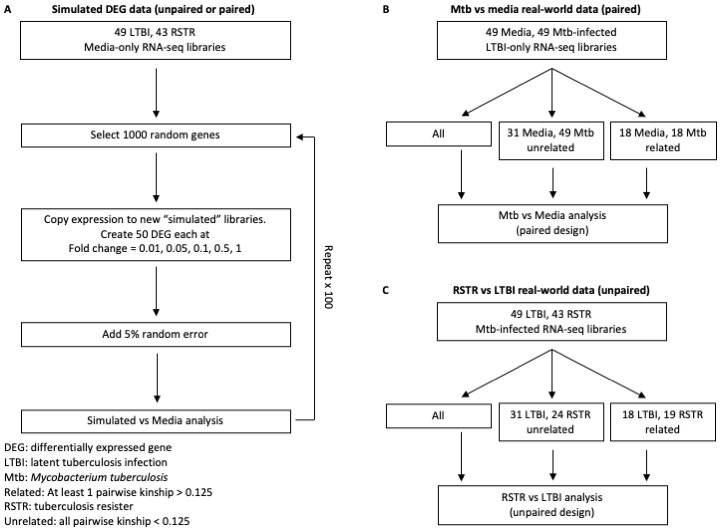


**Figure S1. Dataset sample designs.** (A) Simulated differentially expressed gene (DEG) dataset: 100 simulated datasets were created from the media-only condition samples from two clinical groups, RSTR and LTBI. First, 1000 random genes were selected. Then, DEGs were simulated at fold changes from 1 to 100% and 5% error was added. This was repeated with resampling until 100 datasets were obtained. Models were run for both unpaired and paired designs. (B) Mtb vs media real-world data: RNA-seq data was assessed in 49 LTBI individuals to compare Mtb-infected and media conditions in a paired design. (C) RSTR vs LTBI real-world data: RNA-seq data was assessed in Mtb-infected samples to compare 43 RSTR vs 49 LTBI in an unpaired design. For both (B,C), the entire gene dataset with nearly 14,000 protein-coding genes was used. Unrelated and related subsets were defined at kinship < 0.125 (3rd degree) and assessed separately in linear models.


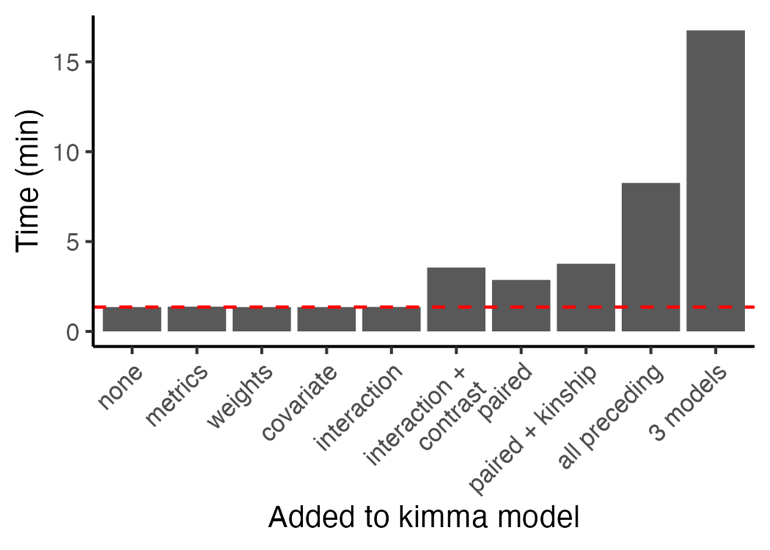


**Figure S2. Time to run kimma with various models on 6 processors.** Horizontal red dashed line indicates the base model of ~condition (Mtb infected vs media) with no additions (none). Sex (female vs male) was used as a covariate and in the interaction term. Contrasts were calculated for all pairwise comparisons of the 4 groups in the condition:sex interaction. The “all preceding” label indicates the model ~condition + sex + condition:sex + kinship + (1|ptID) with all elements to the left including calculating fit metrics and using gene-level weights. The final “3 models” value summarizes running the “all preceding” model under linear, linear mixed effects, and linear mixed effects with kinship modes, yielding results for each of the 3 models.


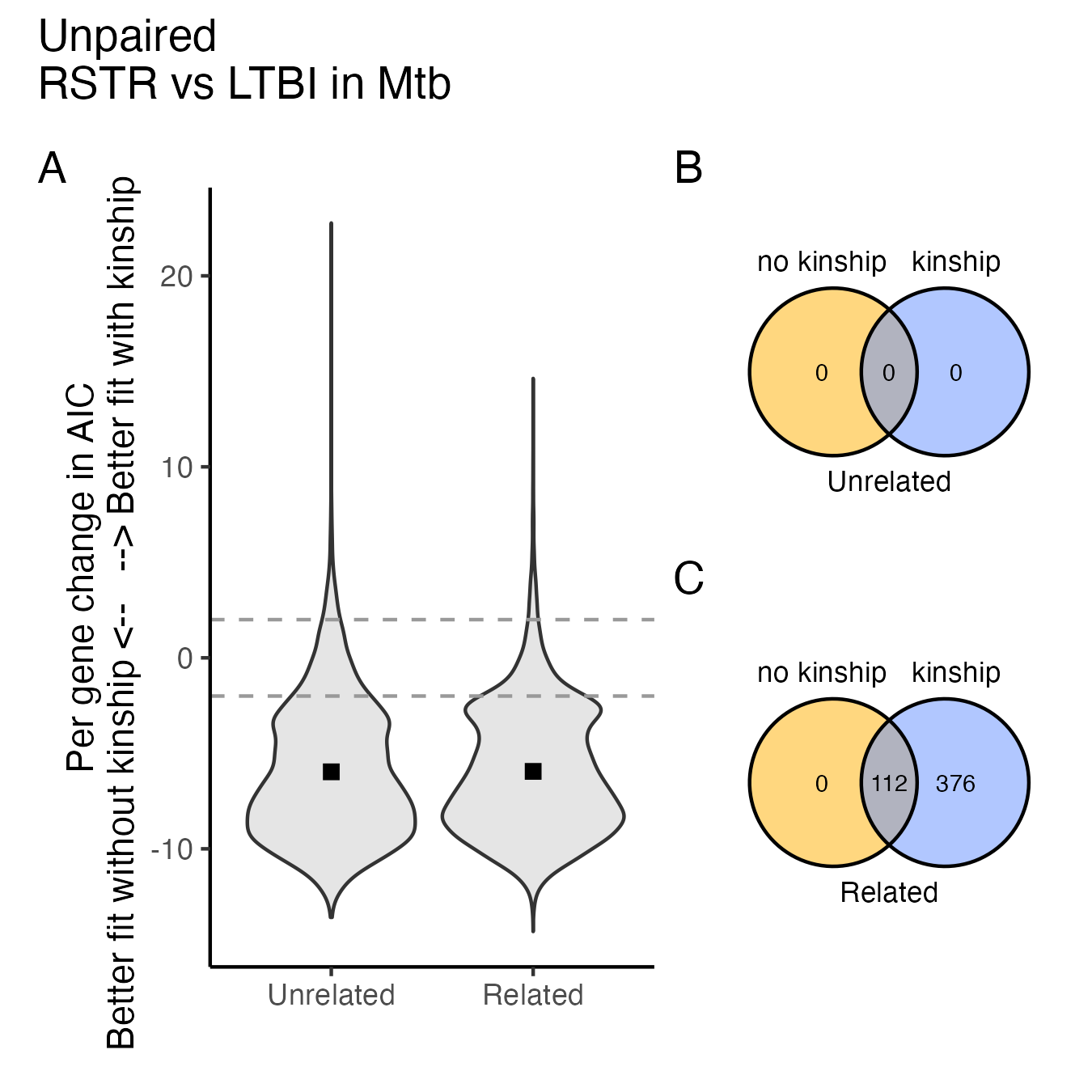


**Figure S3. Unpaired design model fit and DEGs in genetically unrelated and related datasets.** Mtb-infected samples were subset into unrelated and related subsets at a kinship cutoff of 0.125. Subsets were modeled for RSTR vs LTBI expression. (A) Model fit was assessed by AIC . Change in AIC was calculated per gene for the model without kinship minus the model with kinship. Black squares indicate means. Dashed lines indicate minimal change in AIC (y = -2 or 2). DEGs were defined at FDR < 0.05 in the (B) unrelated and (C) related subsets.
